# Acupuncture or Low Frequency Infrared Treatment for Low Back Pain in Chinese Patients: A Discrete Choice Experiment

**DOI:** 10.1371/journal.pone.0126912

**Published:** 2015-05-28

**Authors:** Li-Chia Chen, Li-Jen Cheng, Yan Zhang, Xin He, Roger D. Knaggs

**Affiliations:** 1 Division for Social Research in Medicines and Health, School of Pharmacy, University of Nottingham, Nottingham, United Kingdom; 2 State Key Laboratory of Quality Research in Chinese Medicine, Institute of Chinese Medical Sciences, University of Macau, Macao, China; 3 Pharmacy Practice and Science, College of Pharmacy, University of Iowa, Iowa City, United States of America; 4 Pharmacy Department, Nottingham University Hospitals NHS Trust, Nottingham, United Kingdom; Griffith University, AUSTRALIA

## Abstract

Acupuncture is a popular but controversial treatment option for low back pain. In China, it is practised as traditional Chinese medicine; other treatment strategies for low back pain are commonly practised as Western medicine. Research on patient preference for low back-pain treatment options has been mainly conducted in Western countries and is limited to a willingness-to-pay approach. A stated-preference, discrete choice experiment was conducted to determine Chinese patient preferences and trade-offs for acupuncture and low frequency infrared treatment in low back pain from September 2011 to August 2012 after approval from the Department of Scientific Research in the study settings. Eight-six adult outpatients who visited the ‘traditional medicine department’ at a traditional Chinese medicine hospital and the ‘rehabilitation department’ at a Western medicine hospital in Guangdong Province of China for chronic low back pain during study period participated in an interview survey. A questionnaire containing 10 scenarios (5 attributes in each scenario) was used to ask participants' preference for acupuncture, low frequency infrared treatment or neither option. Validated responses were analysed using a nested-logit model. The decision on whether to receive a therapy was not associated with the expected utility of receiving therapy, female gender and higher out-of-pocket payment significantly decreased chance to receive treatments. Of the utility of receiving either acupuncture or low frequency infrared treatment, the treatment sensation was the most important attribute as an indicator of treatment efficacy, followed by the maximum efficacy, maintenance duration and onset of efficacy, and the out-of-pocket payment. The willingness-to-pay for acupuncture and low frequency infrared treatment were about $618.6 and $592.4 USD per course respectively, demonstrated patients' demand of pain management. The treatment sensation was regarded as an indicator of treatment efficacy and the most important attribute for choosing acupuncture or low frequency infrared treatment. The high willingness-to-pay demonstrated patients' demand of pain management. However, there may be other factors influencing patients' preference to receive treatments.

## Introduction

Back pain is the most commonly reported chronic pain that represents a challenging health and social problem [1, 2]. The prevalence of back pain is comparable worldwide, it has been estimated that 30–40% of the adult population have back pain in a year [3, 4], and a lifetime prevalence of over 60% [3, 5]. Chronic low back pain damages individuals’ physical, psychological and social functioning, causes a large impact on social life and work capability, and leads to a high disease burden to society [6]. In the U.K., the direct cost of treating chronic low back pain was estimated to be £1632 million in 1998, with additional £6650 to £12,300 million indirect costs [7]. In the U.S., it was estimated that 15% to 30% of population suffering from back pain annually, and it is the second leading cause for ambulatory care visits [8, 9].

In Western countries, a multidisciplinary approach, including pharmacological, physical rehabilitation [10], and psychological strategies [11] is generally recommended for managing low back pain. Physiotherapy helps restore movement and function when affected by injury, illness or disability and uses many approaches, including movement and exercise, manual therapy and electrostimulation techniques. However, chronic low back pain is difficult to manage successfully [12], and patients often use complementary or alternative medical treatment options [13, 14]; such as acupuncture [15], massage therapy [16], spinal manipulation [17] and yoga [18], despite current guidance only recommending complementary or alternative medical therapy for those who do not improve with self-care [19].

For example, the U.K. National Institute for Health and Social Care Excellence (NICE) recommends considering a course of acupuncture as an alternative to an exercise programme or manual therapy as the three main therapies for early management of persistent low back pain; a course of acupuncture needling comprising up to a maximum of 10 sessions over a period of up to 12 weeks [20]. Indeed, back pain is the most common reason for using complementary or alternative medicine in the U.S. [21, 22] and Canada [23]. Research on treatment choice and preference for low back pain has suggested the utilisation of complementary or alternative medicine is related to cultural background and accessibility to complementary or alternative medicine in the healthcare system [6, 24].

Acupuncture is a popular but controversial alternative treatment option for low back pain in Western countries [14]. Traditional acupuncture is a holistic approach to improve health, underpinned by the ancient Chinese philosophical and medical theories [25] and based on Chinese medicine diagnosis; whereas Western-style (trigger-point) acupuncture is predominantly practised by doctors and physiotherapists, is based on western medical diagnosis for managing musculoskeletal conditions. Research conducted in Western countries or with participants of non-Asian Chinese ethnicity [26] have found that if there was no concern of out-of-pocket expenses [14], the efficacy of symptom relief rather than the adverse events of acupuncture influenced patients’ willingness to try acupuncture [27], yet acupuncture does not offset the use of other resources [24].

In China, some small surveys have reported that 50% of metropolitan labour workers [28] and 26.2% to 31.5% of soldiers [29, 30] suffered from low back pain. The majority of the tertiary medical facilities in China provide hybrid traditional Chinese medicine and Western medicine services [31], and both pharmacological and non-pharmacological treatments for low back pain can be delivered by both traditional Chinese medicine and Western medicine. Acupuncture is regarded as a traditional Chinese medical strategy in China, whereas other complementary or alternative medicines are mostly derived in Western-style, such as low frequency Infrared therapy, a commonly used physiotherapy treatment for low back pain in China.

Some surveys revealed Chinese people's preference of using traditional Chinese medicine or Western medicine varies with age, gender and disease conditions [32, 33]. Patients' willingness to use acupuncture also changes with age, past treatment experiences, and quality of service delivery [34]. Previous studies have mostly focused on the comparative effectiveness of therapies for low back pain, and less attention has been paid to patient preference; and research on pain management preference were also limited to willingness-to-pay approach [14, 35, 36]. There are no studies examining Chinese patients’ preferences between acupuncture and physiotherapy for the treatment of low back pain.

Stated preference methods are used to elicit an individual’s preferences for ‘alternatives’ expressed in a survey context [37]. Discrete choice experiments are based on a long-standing, well-tested theory of choice behaviour [37] that can be used to investigate consumer preferences for healthcare commodities, the attributes that comprise these commodities and the extent to which individuals are willing to trade-off one attribute against another in making healthcare decisions [37]. Discrete choice experiments are often used to predict demand for healthcare commodities under different scenarios, to assist in the optimal design of commodities to maximize compliance or uptake, and to derive monetary measures of the value of, or willingness to pay for healthcare products and programs, which can potentially be used in cost benefit analysis [37].

Therefore, this study used a stated-preference, discrete choice experiment [38] to elicit Chinese low back pain patient’s preference between acupuncture and low frequency infrared therapy, in order to identify the extent of attributes influencing patients’ choice of treatment, hierarchical importance of these attributes, and patients’ trade-off between risk and benefits for pain management. Low frequency infrared therapy was specified in this study as it was the most commonly used physiotherapy treatment for low back pain in the Western medicine hospitals.

## Method

### Study sample

This study was conducted at outpatient clinics of two tertiary hospitals in Guangdong Province of China. The two hospitals, including one mainly practises traditional Chinese medicine and another Western medicine, are equivalent in size and service capacity, and both practise similar acupuncture and low frequency infrared treatment for managing chronic back pain. Semi-structured face-to-face interviews were conducted from March to November 2010 for exploring attributes and levels to inform the questionnaire design, and then a questionnaire interview survey was conducted from September 2011 to August 2012. The protocol and ethics issues of the study were reviewed and approved by the Department of Scientific Research in the study settings and the Research Committee of the University of Macau as they were the only departments which are responsible for such issues in the research setting at the time, after confirming that this study does not involve invasive, intrusive or potentially harmful procedures and data will be reported and analysed in a confidential and anonymous way.

Adult patients who visited the ‘traditional medicine department’ at the traditional Chinese medicine hospital and the ‘rehabilitation department’ at the Western medicine hospital for chronic low back pain during study period were invited by physicians to participate in the study. Face-to-face interviews were conducted by an on-site researcher (Xin He) when participants were waiting for treatments at the clinics. Participants were informed of the purpose and process of study and ensured the anonymity and confidentiality. All participants were asked for verbal consents as it was suggested by clinical experts and review panels of the Department of Scientific Research that Chinese patients are generally unwilling to give voluntary written consents. Individual’s verbal consent was documented by the researcher on the questionnaire at interview. All the data in the study was anonymized.

### Attributes and levels

Participants were interviewed using a pre-designed questionnaire and asked their preference for pain management in 10 scenarios. Each scenario included attributes with various levels influencing decisions on treatment choices. Attributes and levels were derived from a literature review and were confirmed by a semi-structured interview conducted from March to November 2010 in the research settings using 63 patients who received either acupuncture or low frequency infrared treatment. The five attributes included were: (1) treatment sensation, (2) onset of efficacy, (3) maximum efficacy, (4) duration of efficacy and (5) out-of-pocket payment (Table 1). Participants were given definitions about those attributes by the researcher at interviews.

**Table 1:** Attributes and levels included in the discrete choice experiment.

Attributes	Definition	Level of attributes
Treatment sensation (TRE)	Discomfort caused during treatment	Sore and numb; mild thermal sense and vibration
Onset of efficacy (COU)	Number of courses (6 treatments per course) required to achieve the maximum efficacy	2, 4, 8 courses
Maximum efficacy (IMP)	Maximum pain reduction (ex: ambulatory pain, resting pain, difficulty in doing daily activities or sleep) that can be made by the therapy	Minor, moderate, major improvement
Duration of efficacy (DUR)	Duration of the effect maintenance after treatment	2, 6, 12 months
Out-of-pocket payment (COS)	Out-of-pocket payment on top of insurance coverage required for one course of treatment	120, 600, 1000 CNY per course*

(Note)

*1 Chinese Yuan (CNY) = 0.1575 American Dollar (USD) in August 2012, and 0.1637 in August 2013. The out-of-pocket payment ranked was $18.9, $94.5 and $157.5 USD in August 2012.

Treatment sensation was described as two levels (either ‘sore and num’, replicating the insertion of a needle into skin during acupuncture, or a ‘sense of mild thermal and vibration’, caused by low frequency infrared treatment). Attributes related to treatment effects including the maximum therapeutic efficacy on pain reduction (minor, moderate and major improvement), duration required to attain the maximum effect (calculated as number of courses, and there are 6 treatments per course, generally finished in 3 weeks), and maintenance duration (months) of the therapeutic efficacy were described using three levels. Out-of-pocket payment for a treatment course was also included as three levels to estimate the monetary measure of benefit (willingness-to-pay) for individual attributes (Table 1).

### Pairing scenarios

Combining attributes and levels, a total of 81 therapy profiles (i.e. 3^4^ for the four 3-level attributes) and 3,240 possible pairwise choices (i.e. ${\text{C}}_{2}^{81}=81\times 80÷2)$ emerged from which the attribute ‘treatment sensation’ remained constant in each choice set. Since this number of variables was felt to be too burdensome to participants at interviews [39], a fractional factorial design was developed using an orthogonal matrix to reduce the number of scenarios to manageable levels. Overall, 9 pairs of scenarios from 3^4^ possible profiles and 9 attribute-level combinations for a therapy-option were selected [40], and further tests on the therapy-choice questionnaire concluded that there were equal frequency of attribute levels appearing throughout questionnaire (level balance) and no correlation among the attributes (orthogonality) (S1 Appendix).

**Fig 1:**
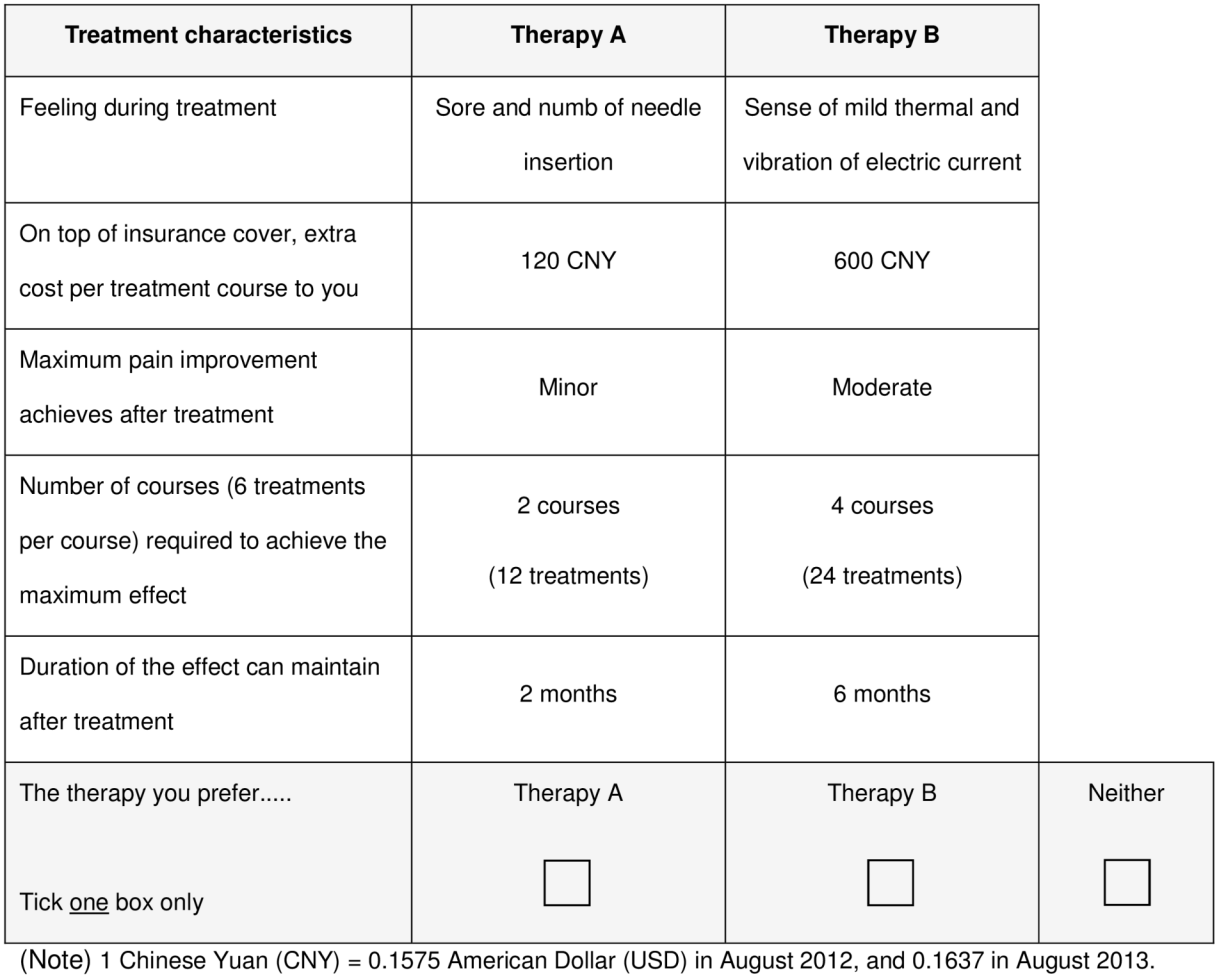
Example of one scenario of choices presented to participants.

For each scenario, participants were presented with three choices, i.e. ‘Therapy A’, ‘Therapy B’, or ‘Neither’ (Fig 1) with clear definitions. Treatment A is acupuncture that uses needles to stimulate trigger points for half an hour. If it is necessary, the needles may connect to electrodes to enhance the efficacy by electric stimulation. Treatment B is low frequency infrared treatment that uses passive low-frequency infrared treatment to the painful area for about half an hour to one hour. Each treatment course includes 6 treatments and is generally finished in 3 weeks. An ‘opt-out’ option was included, as the previous semi-structured interview revealed that low-back pain patients may prefer no treatment or other treatment strategies (such as massage or medication) than acupuncture or low frequency infrared treatment (S1 Fig).

In addition to the 9 included scenarios, one additional scenario was included to test the validity of response (S1 Fig). The validity test scenario included a dominant therapy option that was clearly favoured in the levels of all attributes, and hence should rationally be the preferred option. Responses from participants who failed in the validity test were removed from the analysis. Moreover, participant’s socio-demographics (age, gender, level of education, monthly family income), disease history (intensity of pain) and insurance status (proportion of treatment expense paid by the patient) were also surveyed at the interviews (S1 Fig).

### Piloting

The questionnaire was piloted on 7 patients at the acupuncture department of the traditional Chinese medicine hospital and 8 patients at the rehabilitation department of the Western medicine hospital in June and July 2011. The piloting results showed that the majority of patients understood the instructions and attributes, but occasionally failed to respond to the hypothetical scenarios and applied their own experience instead. Therefore, minor amendments were made to clarify the descriptions of options and instructions of the questionnaire.

### Statistical analysis

Data were analysed using a nested logit model which took account of participants’ joint decision of whether to choose either acupuncture or low frequency infrared treatment and the utility of receiving either therapy, and allowed testing the association between whether to choose a therapy and the (expected) utility of receiving a therapy through the estimation of the inclusive value parameter. Participants’ choices of treatment options and attributes influencing participants’ utility of receiving acupuncture or low frequency infrared treatment were modelled as the following equations:

$$\text{Treated}=\beta_{1}AGE+\;\beta_{2}SEX+\beta_{3}EDU+\beta_{4}INC+\beta_{5}DISC+\beta_{6}PAY_{PROP}+\;\;\mu \;$$ (1)

and

$$v=\delta_{1}TRE+\;\delta_{2}IMP+\delta_{3}COS+\delta_{4}COU+\delta_{5}DUR+\epsilon$$ (2)

The choice of treatment option (Treated) was a binary variable, equalling one when the respondent chose either therapy, and zero if the neither option was chosen. It was modelled as Eq (1) [40], six covariates, including age (AGE), sex (SEX), education level (EDU), monthly family income (INC), the intensity of current back-pain (DISC) and the proportion of expense paid by the patient for current treatment (Pay_Prop) were included to test their influences on choice of treatment option.

The utility associated with receiving therapies (V) was modelled by the determinants of the benefits from two therapies, i.e. the five attributes (TRE, IMP, COS, COU and DUR) defined in Table 1. Regression coefficients β_i_ (*i* = 1,2,3,4,5,6) and δ_j_ (*j* = 1,2,3,4,5) and their corresponding 95% confidence intervals (95%CIs) were calculated, and estimated coefficients were considered statistically significant at P<0.05. ε and μ are the unobservable error terms.

Regression coefficients of Eq (2) [41] quantified the importance of one attribute relative to another on patient’s therapy preference. The greater the size of a (positive) coefficient, the greater utility patients may derive from an increase in the level of that attribute. A marginal rate of substitution between a pair of attributes, i.e. dividing parameter estimates attached to these attributes (e.g.,-$\frac{\delta_{2}}{\delta_{4}}$), represents how much patients are willing to trade the utility gained from one additional unit of one attribute for utility loss from foregoing one unit of another attribute. The willingness-to-pay for non-cost therapy attribute was thus defined as the ratio of the coefficient on the attribute to the coefficient on out-of-pocket cost. All analyses were conducted in STATA version 11.0 (StataCorp LP, College Station, TX).

## Results

### Characteristics of participants

A total of 120 low back pain patients were invited to participate in the survey, including 98 participants from the traditional Chinese medicine hospital and 22 participants from the Western medicine hospital. However, 22 patients who failed in making choices due to either strongly against low frequency infrared treatment (n = 16) or acupuncture (n = 5) or accepted both therapies (n = 1), were not enrolled in the survey. Of the 98 participants who completed the questionnaire, 12 participants were excluded due to failing the validity test (n = 2) or missing socioeconomic data (n = 10), and hence 86 participants were included in analysis. In all, 774 completed choices and 2,322 attribute observations were collected from 86 participants.

Most of the included participants (mean age: 44.5±11.9, range: 22 to 74 years) were female (n = 65; 76%), participated from the traditional Chinese medicine hospitals (n = 70; 81%), and suffered from mild to moderate low back pain (n = 60; 70%). Most participants hold a bachelor or higher degree (n = 56, 65%) and had a monthly family income of 3000 CNY (about $472.4 USD in August 2012) or higher (n = 60, 70%). On average, the out-of-pocket contribution to the total expense of current treatments was about 43% (Table 2).

**Table 2:** Characteristics of the 86 included participants.

Category	Characteristics	Number of participants (%)
Age	Mean age ± standrd deviaiton (range)	44.5±11.9 (22, 74)
Gender	Female	65 (75.6%)
Setting	Chinese medicine hospital (%)	70 (81.4%)
	Western medicine hospital (%)	16 (18.6%)
Out-of-pocket payment	Proportion of out-of-pocket payment to current treatment	43.3% ^(a)^
Education level	Secondary school	2 (2.3%)
	High school and equivalent	28 (32.6%)
	Bachelor	48 (55.8%)
	Post-graduate	8 (9.3%)
Monthly family income	1000 CNY ($157.5 USD) ^(b)^	2 (2.3%)
	1001–3000 CNY ($157.7–472.5 USD)	24 (27.9%)
	3000–5000 CNY ($472.7–787.5 USD)	27 (31.4%)
	>5000 CNY ($787.5 USD)	33 (38.4%)
Intensity of back pain	Mild	27 (31.4%)
	Moderate	33 (38.4%)
	Sevre	16 (18.6%)
	Very severe	10 (11.6%)

(Note)

^(a)^ Proportion of out-of-pocket payment to the cost fo current treatment;

^(b)^ 1 Chinese Yuan (CNY) = 0.1575 American Dollar (USD) in August 2012.

### Influencing factors to choose or opt-out of therapies

Of the 774 completed choices, only 66 (8.5%) were a 'neither' option or alternative therapy, 340 (43.9%) and 368 (47.5%) were acupuncture and low frequency infrared treatment, respectively.

Female (β_2_: -2.15; 95%CI: -3.13, -1.18, P<0.001) and higher proportion of out-of-pocket payment (β_6_: -0.99, 95%CI: -1.84, -0.13; P = 0.024) and higher education levels, especially, bachelor (β_6_: -0.96; 95%CI: -1.69, -0.22) and post-graduate (β_6_: -2.10; 95%CI: -3.39, -0.81) degrees were associated with lower probability of choosing a treatment. In contrast, very severe pain intensity (β_3_: 1.11; 95%CI: -0.05, 2.28) and monthly family income of 3000 to 5000 CNY ($472.4 to $787.4 USD in August 2012) (β_4_: 1.09; 95%CI: 0.32, 1.85) were associated with higher probability of choosing a therapy (Table 3).

**Table 3:** Covariates associated with choosing either therapy in the nested-logit model.

Covariate	Characteristic	Coefficient (95%CI)	P-value
Age (AGE)	Age at interview (year)	0.01 (-0.02, 0.41)	0.444
Gender (SEX)	Female	-2.15 (-3.13, -1.18)	<0.001*
Education level (EDU)	Secondary school	-0.73 (-2.93, 1.48)	0.518
	Bachelor	-0.96 (-1.69, -0.22)	0.011*
	Post-graduate	-2.10 (-3.39, -0.81)	0.001*
Monthly family income (INC)	1000 CNY	0.81 (-1.38, -2.99)	0.468
	3000–5000 CNY	1.09 (0.32, 1.85)	0.005*
	>5000 CNY	0.36 (-0.38, 1.11)	0.336
Intensity of pain (DISC)	Moderate	0.06 (-0.58, 0.69)	0.863
	More sevre	0.36 (-0.48, 1.21)	0.397
	Very severe	1.11 (-0.05, 2.28)	0.061
Out-of-pocket payment (Pay_Pro)	Proportion of out-of-pocket payment	-0.99 (-1.84, -0.13)	0.024*

(Note)

* Statistical significance (p<0.05); CNY: Chinese Yuan (1 CNY = 0.1575 USD in August 2012). Monthly family income rank equalled to $157.5, $472.5–787.5, and >$787.5 USD in August 2012.

Adjusting variables relating to choice of treatment option and the utility associated with receiving therapies, the estimated inclusive value (IV) parameter did not reach statistically significant level (IV: 0.75; 95%CI: 0.39, 1.12; P = 0.388). This indicates that the expected utility of receiving either therapy did not influence patient’s decision on receiving treatment, that is to say, the decision of whether to take up a therapy was made independently of the therapy on offer.

### Impact of attributes on utility

All attributes were found significantly associated with using acupuncture (n = 340) and low frequency infrared treatment (n = 368) for the treatment of low back pain (Table 4). Participants preferred therapy with greater maximum efficacy (δ_2_ for moderate pain reduction: 0.79; 95%CI: 0.38, 1.19; δ_2_ for major pain reduction: 1.65; 95%CI: 0.92, 2.38), longer maintenance duration of efficacy (δ_5_: 0.15; 95%CI: 0.08, 0.22), shorter onset time of efficacy (δ_4_: -0.14; 95%CI: -0.21, -0.07) and lower out-of-pocket payment (δ_3_: -0.00076; 95%CI: -0.00118, -0.00033).

**Table 4:** Impacts of attributes on utility from receiving either therapy in the nested-logit model.

Variable	Coefficient (95%CI)	P-value	Marginal rate of substitution		
			WTP ^(a)^	MRS ^(b)^	
Sore and numb sensation	2.98 (1.00, 4.95)	0.003*	3,928	21 courses	19.48 months
Mild thermal sense and vibration	2.85 (0.87, 4.83)	0.005*	3,762	20 courses	18.66 months
Moderate maximum efficacy	0.79 (0.38, 1.19)	<0.001*	1,039	6 courses	5.16 months
Major maximum efficacy	1.65 (0.92, 2.38)	<0.001*	2,174	12 courses	10.78 months
Out-of-pocket payment	-0.00076 (-0.00118, -0.00033)	<0.001*	Reference	-	-
Onset time of efficacy	-0.14 (-0.21, -0.07)	<0.001*	189	Reference	-
Maintenance duration	0.15 (0.08, 0.22)	<0.001*	202	-	Reference
IV ^(c)^ parameter	0.75 (0.39, 1.12)	0.388			

(Note)

^(a)^ WTP: willingness-to-pay in presented in Chinese Yuan;

^(b)^ MRS: marginal rate of substitution between non-cost attributes;

^(c)^ IV: inclusive value;

* Statistical significance (p<0.05).

It is noteworthy that the treatment sensation, i.e. discomfort signs incurring during treatment were positive for both treatments (δ_1_ for sore and numb sensation: 2.98; 95%CI: 1.00, 4.95; δ_1_ for mild thermal sense and vibration: 2.85; 95%CI: 0.87, 4.83), which suggests that low back pain participants regarded such feelings experiencing during acupuncture and low frequency infrared treatment as a source of satisfaction, rather than discomfort.

### Relative impact of attributes on utility

The relative size of the estimated coefficients implies the importance of the attributes in influencing preferences. The treatment sensation was the most important attribute, followed by the maximum efficacy (pain reduction) expected by the therapy, the maintenance duration of treatment efficacy and the efficacy onset time. The out-of-pocket payment on top of the insurance coverage was the least important attribute (Table 4).

### Trade-off between attributes

The willingness-to-pay for acupuncture therapy (3,928 CNY) and low frequency infrared treatment (3,762 CNY) per course was derived from dividing the estimated coefficients attached to treatment sensation (δ_1_ for sore and numb sensation: 2.98; δ_1_ for mild thermal sense and vibration: 2.85) by the coefficient of out-of-pocket payment (δ_3_: -0.00076). This indicates that participants valued acupuncture by 166 CNY more than low frequency infrared treatment. Comparing the expected maximum efficacy, participants were willing to pay 1,039 CNY and 2,174 CNY more for achieving moderate or major pain reduction compared against minor pain reduction. As for the onset time of efficacy, participants were willing to pay 189 CNY for avoiding one extra treatment course required to achieve the maximum effect. For the maintenance duration, participants were willing to pay 202 CNY for sustaining one extra month of treatment efficacy (Table 4).

The marginal rate of substitution between 'onset time of efficacy' and other attributes demonstrated the threshold number of treatment courses required to achieve the maximum efficacy that participants would accept before switching to another therapy or terminating the therapy was 21 and 20 courses for acupuncture and low frequency infrared treatment. Of the three levels of expected maximum efficacy, threshold numbers of treatment courses required to achieve efficacy were 6 and 12 more courses for achieving moderate and major pain reduction comparing against minor pain reduction. The marginal rate of substitution between 'maintenance duration' and other attributes indicated the minimum duration of treatment efficacy that participants expected were 19.5 months, 18.7 months, 5.2 months and 10.8 months for acupuncture, low frequency infrared treatment, moderate pain reduction and major pain reduction, respectively (Table 4).

## Discussion

This discrete choice experiment found that female gender and higher out-of-pocket payment significantly decreased Chinese low back pain patients’ willingness to receive either acupuncture or low frequency infrared treatment, and their decision on whether to receive a therapy is not associated with the expected utility of therapy. Of the utility from receiving either acupuncture or low frequency infrared treatment, the treatment sensation was the most important attribute, followed by the maximum efficacy, maintenance duration and onset time of efficacy, and the out-of-pocket payment was the least important attribute. The willingness-to-pay for acupuncture and low frequency infrared treatment was 3,928 and 3,762 CNY ($618.6 and $592.4 USD) per course, respectively. Comparing against minor pain reduction, patient expected maximally 6 and 12 courses for achieving moderate and major pain reduction, respectively.

Although the majority of participants were female, they were less willing to choose either treatment. This study consecutively recruited participants in one year study period; the gender composition reflects gender difference in accessing outpatient healthcare in real life. Previous literature has suggested that female low back pain patients tend to report less severe pain [42], but male patients tended to report severe pain which requires more complex interventions such as surgery; and this phenomena was also observed in the prior semi-structured interviews that we conducted before this survey. However, as patients’ treatment choice was not associated with expected utility of either acupuncture or low frequency infrared treatment, there may be other factors influencing patient choice, such as culture or beliefs in traditional Chinese medicine. Previous surveys on the Chinese population suggest those who are elderly (age>60 years), female, metropolitan resident, with higher education, income and knowledge on traditional Chinese medicine were more likely to receive traditional Chinese medicine [32, 33].

Despite the efforts to recruit participants from both traditional Chinese medicine and Western medicine hospitals, this study only included 16 participants (19%) from the Western medicine hospital due to unforeseen challenges. Therefore, it failed to compare patients' choices between traditional Chinese medicine and Western medicine treatment. However, a subgroup analysis conducted on data collected from the Chinese medicine hospital (S1 Table) showed a similar result with the analysis of whole study cohort, and hence the full dataset analysis was reported.

Although this study assumed that discomfort during the treatment may bring disutility to patients and expected negative coefficients, regression on attributes for utility of acupuncture and low frequency infrared treatment revealed positive estimated coefficients on 'treatment sensation', suggesting participants regarded this attribute as 'therapy' itself and derived utility from it. In other words, the role of these coefficients in the model may be alternative-specific constants for two therapies, capturing patient’s potential inherent preference or belief and trust in the therapies, irrespective of therapy attributes.

Previous literature also found that patients' interpretation of response to acupuncture varied with experiences and may also be influenced by culture, expectations and disease conditions [43]. The preliminary semi-structured interview that the attributes in this study were derived from also indicated that patients regard 'pain' or minor reactions at acupuncture as a signal of whether the therapy is working, and the discomfort caused by another therapy as a source of disutility.

Of the three attributes for the clinical efficacy, i.e. onset, maximum pain relief, and duration of effect [44], the maximum pain reduction was regarded as more important than others in this study. Previous studies evaluating stated-preference on patients with various pain conditions [35, 45–48] also demonstrated that pain reduction (or efficacy) was valued as the most important, however the speed of onset was valued more by migraine patients [49–51]. In the preliminary semi-structured interview, patients receiving acupuncture stated that they would try other therapies if satisfactory pain reduction is not achieved in the beginning of therapy. It may be doubtful whether they would consider 6 or 12 additional courses to achieve moderate or major pain reduction. An explanation is participants were willing to receive courses when treatment efficacy is guaranteed, as explicitly described in the options.

In addition, as the two study hospitals are tertiary medical facilities, the treatments may be more expensive than other medical facilities in China. Most low back pain participants received treatment under some forms of insurance coverage; therefore they were under lesser financial pressure. However, the willingness-to-pay for acupuncture and low frequency infrared treatment was estimated to be 3,928 and 3,762 CNY per course, that is higher than an individual's monthly income which is estimated to be 2518.23 CNY (the annual income per person was 30218.76 CNY in Guangdong Province in 2011), this reflects healthcare demands of low back pain patients.

To our knowledge, this study is the first to elicit Chinese low back pain patient’s preference for acupuncture and low frequency infrared treatment using a discrete choice experiment. Kløjgaard et al. (2014) reported a discrete choice experiment conducted in a Danish Spine Centre to quantify utilities and trade-off treatment outcomes between surgical and non-surgical strategies. Unsurprisingly, majority of the respondents prefer nonsurgical interventions, but patients are willing to wait for more ideal outcomes and preferred interventions. The attributes for eliciting patients’ preference between invasive surgery and conservative non-surgical treatments, including risk of relapse, reduction in pain, and expected increase in the ability to perform activities of daily living, are different from our study that explored choices between non-surgical treatments, and hence the results are not comparable [52].

However, there are several limitations with this study. The recruitment of participants is a major challenge for conducting survey studies in China. Participants who consented to this study in the two hospitals are mostly female and generally well educated, hence are not representative of the general low back pain patients in China. Due to unforeseen reasons, this study only included 16 participants from the Western medicine hospital. In addition, the study was conducted in the Guangzhou city in Guangdong Province, where the population historically more favour traditional Chinese medicine. Patients visiting outpatient departments in hospitals may demand advanced healthcare and be more willing to be interviewed, their preference and demands may differ from patients who did not access hospitals as their pain intensity and willingness-to-pay may vary. Anecdotal evidence also suggested that Chinese patients are likely to receive both traditional Chinese medicine and Western medicine at the same time, therefore in addition to neither option, a 'take-both' option may need to be considered in the future. Finally, the definitions of treatment options in this study were based on the conventional practices in both hospitals; it may be different to the conventional practices in Western countries.

## Conclusion

Chinese low back pain patients regard the treatment sensation was the most important source of utility of receiving acupuncture or low frequency infrared treatment, followed by the maximum efficacy, maintenance duration and onset time of efficacy, and out-of-pocket payment. The sensation during treatment was regarded as a source of satisfaction. The high willingness-to-pay demonstrated low back pain patients' demand of pain management. However, there may be other factors influencing patients' preference to receive treatments.

## Supporting Information

S1 Appendix Pairing scenarios in questionnaire design. (DOCX)

S1 Fig Questionnaire for interviews. (DOCX)

S1 Table Impacts of attributes on utility from receiving either therapy in the nested-logit model of participants recruited from Chinese medicine hospital. (DOCX)
