# Supplementary material for: Acupuncture or Low Frequency Infrared Treatment for Low Back Pain in Chinese Patients: A Discrete Choice Experiment
Source: PLoS One. 2015 May 28;10(5):e0126912. doi: 10.1371/journal.pone.0126912 (PMC4447362; doi:10.1371/journal.pone.0126912)
Supplement: S1 Appendix — (DOCX) [file pone.0126912.s001.docx]

Appendix. Pairing scenarios in questionnaire design

According to the test from <http://support.sas.com/techsup/technote/ts723_Designs.txt> , since four of the six attributes are three levels, the starting design is:

3^4 n=9

0000

0121

0212

1022

1110

1201

2011

2102

2220

The main results are:

**Main Results**

Number of choicesets: 9
Det C is: 5.40269e-17
Main effects are uncorrelated
Efficiency compared with optimal design for choice set size m = 2: 100.00000%
Efficiency compared with optimal design for optimal choice set size m = 3: 75.00000%

**Choice sets**

0 0 0 0 1 1 1 1

0 1 2 1 1 2 0 2

0 2 1 2 1 0 2 0

1 0 2 2 2 1 0 0

1 1 1 0 2 2 2 1

1 2 0 1 2 0 1 2

2 0 1 1 0 1 2 2

2 1 0 2 0 2 1 0

2 2 2 0 0 0 0 1

**Contrast matrix B**

-1/(3*√(6)) -1/(3*√(6)) -1/(3*√(6)) -1/(3*√(6)) -1/(3*√(6)) -1/(3*√(6)) 0 0 0 0 0 0 1/(3*√(6)) 1/(3*√(6)) 1/(3*√(6)) 1/(3*√(6)) 1/(3*√(6)) 1/(3*√(6))

1/(9*√(2)) 1/(9*√(2)) 1/(9*√(2)) 1/(9*√(2)) 1/(9*√(2)) 1/(9*√(2)) -√(2)/9 -√(2)/9 -√(2)/9 -√(2)/9 -√(2)/9 -√(2)/9 1/(9*√(2)) 1/(9*√(2)) 1/(9*√(2)) 1/(9*√(2)) 1/(9*√(2)) 1/(9*√(2))

-1/(3*√(6)) -1/(3*√(6)) 0 0 1/(3*√(6)) 1/(3*√(6)) -1/(3*√(6)) -1/(3*√(6)) 0 0 1/(3*√(6)) 1/(3*√(6)) -1/(3*√(6)) -1/(3*√(6)) 0 0 1/(3*√(6)) 1/(3*√(6))

1/(9*√(2)) 1/(9*√(2)) -√(2)/9 -√(2)/9 1/(9*√(2)) 1/(9*√(2)) 1/(9*√(2)) 1/(9*√(2)) -√(2)/9 -√(2)/9 1/(9*√(2)) 1/(9*√(2)) 1/(9*√(2)) 1/(9*√(2)) -√(2)/9 -√(2)/9 1/(9*√(2)) 1/(9*√(2))

-1/(3*√(6)) -1/(3*√(6)) 1/(3*√(6)) 1/(3*√(6)) 0 0 1/(3*√(6)) 1/(3*√(6)) 0 0 -1/(3*√(6)) -1/(3*√(6)) 0 0 -1/(3*√(6)) -1/(3*√(6)) 1/(3*√(6)) 1/(3*√(6))

1/(9*√(2)) 1/(9*√(2)) 1/(9*√(2)) 1/(9*√(2)) -√(2)/9 -√(2)/9 1/(9*√(2)) 1/(9*√(2)) -√(2)/9 -√(2)/9 1/(9*√(2)) 1/(9*√(2)) -√(2)/9 -√(2)/9 1/(9*√(2)) 1/(9*√(2)) 1/(9*√(2)) 1/(9*√(2))

-1/(3*√(6)) 0 0 1/(3*√(6)) -1/(3*√(6)) 1/(3*√(6)) -1/(3*√(6)) 1/(3*√(6)) -1/(3*√(6)) 0 0 1/(3*√(6)) 0 1/(3*√(6)) -1/(3*√(6)) 1/(3*√(6)) -1/(3*√(6)) 0

1/(9*√(2)) -√(2)/9 -√(2)/9 1/(9*√(2)) 1/(9*√(2)) 1/(9*√(2)) 1/(9*√(2)) 1/(9*√(2)) 1/(9*√(2)) -√(2)/9 -√(2)/9 1/(9*√(2)) -√(2)/9 1/(9*√(2)) 1/(9*√(2)) 1/(9*√(2)) 1/(9*√(2)) -√(2)/9

**Λ matrix**

1/36 0 0 0 0 0 0 0 0 -1/36 0 0 0 0 0 0 0 0

0 1/36 0 0 0 0 0 0 0 0 0 0 0 0 0 0 -1/36 0

0 0 1/36 0 0 0 0 0 0 0 0 -1/36 0 0 0 0 0 0

0 0 0 1/36 0 0 0 0 0 0 0 0 -1/36 0 0 0 0 0

0 0 0 0 1/36 0 0 0 0 0 0 0 0 0 0 -1/36 0 0

0 0 0 0 0 1/36 -1/36 0 0 0 0 0 0 0 0 0 0 0

0 0 0 0 0 -1/36 1/36 0 0 0 0 0 0 0 0 0 0 0

0 0 0 0 0 0 0 1/36 0 0 0 0 0 0 -1/36 0 0 0

0 0 0 0 0 0 0 0 1/36 0 0 0 0 0 0 0 0 -1/36

-1/36 0 0 0 0 0 0 0 0 1/36 0 0 0 0 0 0 0 0

0 0 0 0 0 0 0 0 0 0 1/36 0 0 -1/36 0 0 0 0

0 0 -1/36 0 0 0 0 0 0 0 0 1/36 0 0 0 0 0 0

0 0 0 -1/36 0 0 0 0 0 0 0 0 1/36 0 0 0 0 0

0 0 0 0 0 0 0 0 0 0 -1/36 0 0 1/36 0 0 0 0

0 0 0 0 0 0 0 -1/36 0 0 0 0 0 0 1/36 0 0 0

0 0 0 0 -1/36 0 0 0 0 0 0 0 0 0 0 1/36 0 0

0 -1/36 0 0 0 0 0 0 0 0 0 0 0 0 0 0 1/36 0

0 0 0 0 0 0 0 0 -1/36 0 0 0 0 0 0 0 0 1/36

**Information matrix C**

1/108 0 0 0 0 0 0 0

0 1/108 0 0 0 0 0 0

0 0 1/108 0 0 0 0 0

0 0 0 1/108 0 0 0 0

0 0 0 0 1/108 0 0 0

0 0 0 0 0 1/108 0 0

0 0 0 0 0 0 1/108 0

0 0 0 0 0 0 0 1/108

**Variance-Covariance matrix C^-1^**

108 0 0 0 0 0 0 0

0 108 0 0 0 0 0 0

0 0 108 0 0 0 0 0

0 0 0 108 0 0 0 0

0 0 0 0 108 0 0 0

0 0 0 0 0 108 0 0

0 0 0 0 0 0 108 0

0 0 0 0 0 0 0 108

**Correlation matrix**

1. 0 0 0 0 0 0 0

0 1. 0 0 0 0 0 0

0 0 1. 0 0 0 0 0

0 0 0 1. 0 0 0 0

0 0 0 0 1. 0 0 0

0 0 0 0 0 1. 0 0

0 0 0 0 0 0 1. 0

0 0 0 0 0 0 0 1.

**End of output.**
