# Supplementary material for: Acupuncture or Low Frequency Infrared Treatment for Low Back Pain in Chinese Patients: A Discrete Choice Experiment
Source: PLoS One. 2015 May 28;10(5):e0126912. doi: 10.1371/journal.pone.0126912 (PMC4447362; doi:10.1371/journal.pone.0126912)
Supplement: S1 Fig — (DOCX) [file pone.0126912.s002.docx]

S1 Fig. Questionnaire for interviews

Survey Questionnaire for Outpatients’ Preference on Acupuncture or Low Frequency Infrared Treatment for Low Back Pain - Guangdong Province Traditional Chinese Medicine Hospital

| Thank you for taking time to participant this research. This questionnaire survey aims to understand the reasons for why you choose or do not choose the following treatment, in order to enhance more satisfactory medical services in the future. "Lower back pain" refers to pain occurs on your back from the rib to hip area, with or without lower extremities symptoms. It can be managed by the following two treatments:  **Treatment A** is acupuncture that uses needles to stimulate trigger points for controlling pain. There may be a slight pain when the needle inserts the skin, in addition you may feel sore, numb, tingling and swelling on the insertion sites. If it is necessary, the needles may connect to electrodes to enhance the efficacy by electric stimulation. It may also cause minor bleeding when withdrawing needles. The treatment takes about half an hour. Insertion of needle is conducted by physicians while adjusting electric current and managing bleeding needle are conducted by interns or resident physicians.  **Treatment B** is low frequency infrared treatment that uses low-frequency, infrared, low-frequency instrument to control pain. During the treatment, you may fell slightly warm and mild electric current stimulation. The treatment takes about half an hour to one hour, and is normally provided by interns or resident physicians.  Now, I would like to present you with 10 scenarios. Please consider following five aspects describing the efficacy of treatment and price, and select the treatments that you prefer. Both treatment courses include six times of treatment.  The first aspect refers to how the two treatments feel, ‘sore and numb’, replicating the insertion of a needle into skin during acupuncture, and a ‘sense of mild thermal and vibration’, caused by low frequency infrared treatment. The second aspect estimates how much extra you will need to pay per treatment course on top of insurance cover. The third aspect is the best efficacy that the treatment can achieve in pain reduction which can be categorised in minor, moderate and major improvement. The minor improvement refers to slight improvement in pain related discomforts and inconvenience, but no great pain relief. Moderate improvement refers to most but not completely relief of discomfort. Major improvement refers to your totally satisfy with the pain management. The forth aspect refers to how many times of treatment you need to achieve the satisfactory effect. It is calculated as number of courses, and there are 6 treatments per course, generally finished in 3 weeks), and maintenance duration (months) of the therapeutic efficacy were described using three levels. The fifth aspect refers to how long the treatment effect can be maintained. |
| --- |

**Scenario 1**

| **Treatment characteristics** | **Therapy A** | **Therapy B** |  |
| --- | --- | --- | --- |
| Feeling during treatment | Sore and numb of needle insertion | Sense of mild thermal and vibration of electric current |  |
| On top of insurance cover, extra cost per treatment course to you | 120 CNY | 600 CNY |  |
| Maximum pain improvement achieves after treatment | Minor | Moderate |  |
| Number of courses (6 treatments per course) required to achieve the maximum effect | 2 courses  (12 treatments) | 4 courses  (24 treatments) |  |
| Duration of the effect can maintain after treatment | 2 months | 6 months |  |
| The therapy you prefer.....  Tick one box only | Therapy A | Therapy B | Neither |

**Scenario 2**

| **Treatment characteristics** | **Therapy A** | **Therapy B** |  |
| --- | --- | --- | --- |
| Feeling during treatment | Sore and numb of needle insertion | Sense of mild thermal and vibration of electric current |  |
| On top of insurance cover, extra cost per treatment course to you | 120 CNY | 600 CNY |  |
| Maximum pain improvement achieves after treatment | Moderate | Major |  |
| Number of courses (6 treatments per course) required to achieve the maximum effect | 8 courses  (48 treatments) | 2 courses  (12 treatments) |  |
| Duration of the effect can maintain after treatment | 6 months | 12 months |  |
| The therapy you prefer.....  Tick one box only | Therapy A | Therapy B | Neither |

**Scenario 3**

| **Treatment characteristics** | **Therapy A** | **Therapy B** |  |
| --- | --- | --- | --- |
| Feeling during treatment | Sore and numb of needle insertion | Sense of mild thermal and vibration of electric current |  |
| On top of insurance cover, extra cost per treatment course to you | 120 CNY | 600 CNY |  |
| Maximum pain improvement achieves after treatment | Major | Minor |  |
| Number of courses (6 treatments per course) required to achieve the maximum effect | 4 courses  (24 treatments) | 8 courses  (48 treatments) |  |
| Duration of the effect can maintain after treatment | 12 months | 2 months |  |
| The therapy you prefer.....  Tick one box only | Therapy A | Therapy B | Neither |

**Scenario 4**

| **Treatment characteristics** | **Therapy A** | **Therapy B** |  |
| --- | --- | --- | --- |
| Feeling during treatment | Sore and numb of needle insertion | Sense of mild thermal and vibration of electric current |  |
| On top of insurance cover, extra cost per treatment course to you | 600 CNY | 1000 CNY |  |
| Maximum pain improvement achieves after treatment | Minor | Moderate |  |
| Number of courses (6 treatments per course) required to achieve the maximum effect | 8 courses  (48 treatments) | 2 courses  (12 treatments) |  |
| Duration of the effect can maintain after treatment | 12 months | 2 months |  |
| The therapy you prefer.....  Tick one box only | Therapy A | Therapy B | Neither |

**Scenario 5**

| **Treatment characteristics** | **Therapy A** | **Therapy B** |  |
| --- | --- | --- | --- |
| Feeling during treatment | Sore and numb of needle insertion | Sense of mild thermal and vibration of electric current |  |
| On top of insurance cover, extra cost per treatment course to you | 600 CNY | 1000 CNY |  |
| Maximum pain improvement achieves after treatment | Moderate | Major |  |
| Number of courses (6 treatments per course) required to achieve the maximum effect | 4 courses  (24 treatments) | 8 courses  (48 treatments) |  |
| Duration of the effect can maintain after treatment | 2 months | 6 months |  |
| The therapy you prefer.....  Tick one box only | Therapy A | Therapy B | Neither |

**Scenario 6**

| **Treatment characteristics** | **Therapy A** | **Therapy B** |  |
| --- | --- | --- | --- |
| Feeling during treatment | Sore and numb of needle insertion | Sense of mild thermal and vibration of electric current |  |
| On top of insurance cover, extra cost per treatment course to you | 600 CNY | 1000 CNY |  |
| Maximum pain improvement achieves after treatment | Major | Minor |  |
| Number of courses (6 treatments per course) required to achieve the maximum effect | 2 courses  (12 treatments) | 4 courses  (24 treatments) |  |
| Duration of the effect can maintain after treatment | 6 months | 12 months |  |
| The therapy you prefer.....  Tick one box only | Therapy A | Therapy B | Neither |

**Scenario 7**

| **Treatment characteristics** | **Therapy A** | **Therapy B** |  |
| --- | --- | --- | --- |
| Feeling during treatment | Sore and numb of needle insertion | Sense of mild thermal and vibration of electric current |  |
| On top of insurance cover, extra cost per treatment course to you | 1000 CNY | 120 CNY |  |
| Maximum pain improvement achieves after treatment | Minor | Moderate |  |
| Number of courses (6 treatments per course) required to achieve the maximum effect | 4 courses  (24 treatments) | 8 courses  (48 treatments) |  |
| Duration of the effect can maintain after treatment | 6 months | 12 months |  |
| The therapy you prefer.....  Tick one box only | Therapy A | Therapy B | Neither |

**Scenario 8**

| **Treatment characteristics** | **Therapy A** | **Therapy B** |  |
| --- | --- | --- | --- |
| Feeling during treatment | Sore and numb of needle insertion | Sense of mild thermal and vibration of electric current |  |
| On top of insurance cover, extra cost per treatment course to you | 1000 CNY | 120 CNY |  |
| Maximum pain improvement achieves after treatment | Moderate | Major |  |
| Number of courses (6 treatments per course) required to achieve the maximum effect | 2 courses  (12 treatments) | 4 courses  (24 treatments) |  |
| Duration of the effect can maintain after treatment | 12 months | 2 months |  |
| The therapy you prefer.....  Tick one box only | Therapy A | Therapy B | Neither |

**Scenario 9**

| **Treatment characteristics** | **Therapy A** | **Therapy B** |  |
| --- | --- | --- | --- |
| Feeling during treatment | Sore and numb of needle insertion | Sense of mild thermal and vibration of electric current |  |
| On top of insurance cover, extra cost per treatment course to you | 1000 CNY | 120 CNY |  |
| Maximum pain improvement achieves after treatment | Major | Minor |  |
| Number of courses (6 treatments per course) required to achieve the maximum effect | 8 courses  (48 treatments) | 2 courses  (12 treatments) |  |
| Duration of the effect can maintain after treatment | 2 months | 6 months |  |
| The therapy you prefer.....  Tick one box only | Therapy A | Therapy B | Neither |

**Scenario 10**

| **Treatment characteristics** | **Therapy A** | **Therapy B** |  |
| --- | --- | --- | --- |
| Feeling during treatment | Sore and numb of needle insertion | Sense of mild thermal and vibration of electric current |  |
| On top of insurance cover, extra cost per treatment course to you | 1000 CNY | 120 CNY |  |
| Maximum pain improvement achieves after treatment | Minor | Major |  |
| Number of courses (6 treatments per course) required to achieve the maximum effect | 8 courses  (48 treatments) | 2 courses  (12 treatments) |  |
| Duration of the effect can maintain after treatment | 2 months | 12 months |  |
| The therapy you prefer.....  Tick one box only | Therapy A | Therapy B | Neither |

| **Personal Information**  It is much appreciated if you are willing to provide your personal information on education level, occupation and salary and other relevant information to assist the analysis of this interview. All the information will be analyzed and reported anonymously. You have the right to withdraw your consents on providing those data at any time. | | | | | | | | | | | | | | | | | | | | | | | | | | | | | | | | | |  |
| --- | --- | --- | --- | --- | --- | --- | --- | --- | --- | --- | --- | --- | --- | --- | --- | --- | --- | --- | --- | --- | --- | --- | --- | --- | --- | --- | --- | --- | --- | --- | --- | --- | --- | --- |
| Gender | 1. Male | □ | 2. Female | | | | | | | | | □ | | | |  | | | Age: | | | | |  | | | |  | |  | Year | | |  |
| Occupation | 1. Labour worker | | | □ | | | 1. Office worker | | | | | | | | | | | | | □ | | | | | 1. Technical worker | | | | | | | □ | |  |
|  | 1. Academia | | | □ | | | 1. Student | | | | | | | | | | | | | □ | | | | | 1. Other | | | | | | | □ | |  |
| Education level | 1. Secondary school | | | | | | | | | □ | | | | 1. High school and equivalent | | | | | | | | | | | | | | | | | | □ | |  |
|  | 1. Bachelor | | | | | | | | | □ | | | | 1. Post-graduate | | | | | | | | | | | | | | | | | | □ | |  |
| Monthly family income | 1. 1000 CNY | | | | | | | | | □ | | | | 1. 1001-3000 CNY | | | | | | | | | | | | | | | | | | □ | |  |
|  | 1. 3000-5000 CNY | | | | | | | | | □ | | | | 1. >5000 CNY | | | | | | | | | | | | | | | | | | □ | |  |
| Low back pain diagnosis | 1. Intervertebral disc herniation, hyperosteogeny of lumbar vertebrae | | | | | | | | | | | | □ | | | | | 1. lumbar muscle strain | | | | | | | | | | | | | | □ | |  |
|  | 1. Sprain or injury | | | | | | | | | | | | □ | | | | | 1. Postpartum lumbar pain | | | | | | | | | | | | | | □ | |  |
|  | 1. Other lumbar pain   (please specify) | | | | | | | | | | | |  | | | | | 1. Unknown reason | | | | | | | | | | | | | | □ | |  |
| Disease history | 1. < 3 months | | | | □ | | | 1. 3-12 months | | | | | | | | | | | | | | □ | | | | | 1. > one year | | | | | □ | |  |
| Pain related functionality | 1. Sitting | | | | □ | | | 1. Walking | | | | | | | | | | | | | □ | | | | | 1. Squat | | | | | | □ | |  |
|  | 1. Bend | | | | □ | | | 1. Lift | | | | | | | | | | | | | □ | | | | | 1. Housework | | | | | | □ | |  |
|  | 1. Working | | | | □ | | | 1. Sleeping | | | | | | | | | | | | | □ | | | | | 1. Childcare | | | | | | □ | |  |
|  | 1. Other: | | | | | | | | | | | | | | | | | | | | | | | | | | | | | | | | |  |
| Pain intensity |  | | | | | | | | | | | | | | | | | | | | | | | | | | | | | | | | |  |
|  | 1. Mild | | | | | 1. Moderate | | | | | | | | | | | 1. Severe | | | | | | | | | | | | 1. Very severe | | | | |  |
|  | □ | | | | | □ | | | | | | | | | | | □ | | | | | | | | | | | | □ | | | | |  |
| Previous treatment (multiple choice) | 1. Western medicine | | | | | | | | | | □ | | | | 1. Chinese medicine | | | | | | | | | | | | | | | | | | □ | |
|  | 1. Low Frequency Infrared Treatment | | | | | | | | | | □ | | | | 1. Acupuncture | | | | | | | | | | | | | | | | | | □ | |
|  | 1. Massage | | | | | | | | | | □ | | | | 1. Cupping | | | | | | | | | | | | | | | | | | □ | |
|  | 1. Surgery | | | | | | | | | | □ | | | | 1. Other: | | | | | | | | | | | | | | | | | | □ | |
| Healthcare payment | 1. Public insurance □ | | | | | | | | copayment % | | | | | | | | | | | | | | Contracted hospital? □ | | | | | | | | | | |  |
|  | 1. Health insurance □ | | | | | | | | copayment % | | | | | | | | | | | | | | Contracted hospital? □ | | | | | | | | | | |  |
|  | 1. Out-of-pocket □ | | | | | | | |  | | | | | | | | | | | | | | | | | | | | | | | | |  |
