# Supplementary material for: Acupuncture or Low Frequency Infrared Treatment for Low Back Pain in Chinese Patients: A Discrete Choice Experiment
Source: PLoS One. 2015 May 28;10(5):e0126912. doi: 10.1371/journal.pone.0126912 (PMC4447362; doi:10.1371/journal.pone.0126912)
Supplement: S1 Table — (DOCX) [file pone.0126912.s003.docx]

S1 Table. Impacts of attributes on utility from receiving either therapy in the nested-logit model of participants recruited from Chinese medicine hospital

| **Variable** | **Coefficient (95%CI)** | **P-value** |
| --- | --- | --- |
|  |  |  |
| Sore and numb sensation | 3.81 (1.45, 6.17) | 0.002* |
| Mild thermal sense and vibration | 3.64 (1.27, 6.02) | 0.003* |
| Moderate maximum efficacy | 0.65 (0.24, 1.06) | 0.002* |
| Major maximum efficacy | 1.42 (0.64, 2.21) | <0.001* |
| Out-of-pocket payment | -0.00073 (-0.00122, -0.00023) | 0.004* |
| Onset time of efficacy | -0.14 (-0.24, -0.06) | 0.001* |
| Maintenance duration | 0.15 (0.07, 0.24) | <0.001* |

(Note) * Statistical significance (p<0.05).
